# Seroprevalence of Epstein-Barr virus, hepatitis B virus, hepatitis C virus, and human immunodeficiency virus among patients with non-Hodgkin's lymphoma at the Yaounde General Hospital, Cameroon

**DOI:** 10.11604/pamj.2022.42.58.31473

**Published:** 2022-05-19

**Authors:** Jacky Njiki Bikoï, Esther Del Florence Moni Ndedi, Etienne Atenguena Okobalemba, Donatien Serge Mbaga, Chris André Mbongue Mikangue, Alexandra Emmanuelle Membangbi, Elsa Makue Nguiffo, Justin Olivier Essindi, George Ikomey Mondinde, Sara Honorine Riwom Essama

**Affiliations:** 1 Faculty of Science, Laboratory of Microbiology University of Yaoundé, Yaoundé, Cameroon,; 2 Faculty of Medicine and Biomedical Sciences, University of Yaoundé, Yaoundé, Cameroon,; 3 Department of Medical Oncology Unit, Yaoundé General Hospital, Yaoundé, Cameroon,; 4 Faculty of Medicine and Biomedical Sciences, Centre for Study and Control of Communicable Diseases, University of Yaoundé, Yaoundé, Cameroon

**Keywords:** Non-Hodgkin lymphoma, virus, seroprevalence, Epstein-Barr virus, hepatitis B virus, hepatitis C virus, human immunodeficiency virus

## Abstract

The non-Hodgkin's lymphomas (NHLs) are a diverse group of malignancies that originate in lymphoid cells, heterogeneous in clinical behavior, morphology, cellular origin, etiology, and pathogenesis. A viral infectious etiology had been associated with them. This study aimed to investigate the seroprevalence of Epstein-Barr virus (EBV), Hepatitis B virus (HBV), Hepatitis C virus (HCV) and Human Immunodeficiency Virus (HIV) among patients with NHL at the Yaoundé General Hospital (YGH). Participants for this cross-sectional study were recruited at the medical oncology unit from October 2018 to December 2019. For each patient fulfilling the inclusion criteria, five milliliters of blood were drawn at the crook of their elbows in EDTA tubes. Then, EBV, HIV, HBV, and HVC screening were done using the Rapid Diagnostic Tests (RDTs); Bio-Rad EBV, Alere Determine HIV-1/HIV-2, HBV the best diagnostic and HVC Wondfo biotech respectively. Participants were made up of sixty-three males (69.23%) and twenty-eight females (30.77%). Their ages ranged from nineteen to seventy-eight years, with a mean ± SD of 56.5 ± 15.5. There were eight HIV patients (8.8%) followed by five EBV or HBV patients (5.5%). Three patients were coinfected with HIV+EBV (3.3%) while only two patients (2.2%) had HCV. Only HIV and EBV were seen coinfected. The presence of HBV and HCV in patients with NHL reveals the need to understand how these viruses induce lymphoproliferative diseases, more precisely, the non-Hodgkin´s lymphoma.

## Introduction

The non-Hodgkin's lymphomas (NHLs) are a diverse group of malignancies with a common origin in lymphoid cells [1]. They are heterogeneous in clinical behavior, morphologic appearance, cellular origin, etiology and pathogenesis [2]. The aetiology of NHL has been the subject of ongoing investigations [3]. Risks for developing NHL include immunosuppression and the effect of infectious agents like *Herpesviridae* such as Epstein-Barr virus (EBV); Human T-Lymphotropic virus 1 (HTLV-1) and Human Herpesvirus 8 (HHV-8) [4]. Other infectious agents found associated with lymphoma include Human Immunodeficiency Virus (HIV); Hepatitis B virus (HBV); Hepatitis C virus (HCV); *Helicobacter pylori; Chlamydia psittaci, Borrelia burgdorferi* [5]. Each of these infectious agents develops preferentially in one type of NHL. Overall, in African countries prevalence fluctuates between 4.2% and 7.6% According to the data from the World Health Organization International Agency for Research on Cancer (IARC), the NHL rate is rising worldwide and is higher in developed countries [6]. The study aimed at investigating the seroprevalence of EBV, HBV, HCV and HIV among patients with NHL at the Yaoundé General Hospital (YGH).

## Methods

**Study design and settings:** for this cross-sectional study, were recruited in the Medical Oncology unit of Yaoundé General Hospital, all patients with NLH who come to meet the physician from 1^st^ October 2018 to 31^st^ December 2019. An interviewer-based standard questionnaire was administered to all participants to obtain demographic data as well as information on age, sex, marital status, alcohol drinking, and smoking. We also investigated the knowledge of our participants about the EBV, HBV, HCV and HIV transmission routes, risk factors, and preventive measures.

**Sample size and eligibility criteria:** the sample size was estimated using the formula described by *Charan* and *Biswas* [7], using the prevalence of NHL in Cameroon [3]. The inclusion criterion was any patient with NHL who met the medical doctor during the study period. The non-inclusion criteria were; 1) all patients treated with immunosuppressing drugs and corticotherapy drugs; 2) patients unable to give their consent, 3) patients under 18 years of age. Ninety-one (91) patients fulfilled inclusion criteria and were recorded. Thirty-five of them had not started treatment. Fifty-six were going through chemotherapy. The chemotherapy used in this oncology unit for NHL patients was Cyclophosphamide, doxorubicine, Vincristine and Prednisone (CHOP).

**Sampling and plasma testing:** for each patient, five milliliters of blood were drawn at the crook of their elbows in Acid ethylene diamine tetra acetic (EDTA) tubes. The samples were coded and carried to the Centre for Study and Control of Communicable Diseases at the Faculty of Medicine and Biomedical Sciences. Then, samples were centrifuged at 1000g for 5 minutes to obtain plasma, after that, plasma was divided into two aliquots. The first aliquot was used to screen EBV, HIV, HBV, and HVC by using rapid diagnostic tests (RDTs) Bio-rad EBV, Alere Determine HIV-1/HIV-2, HBV best diagnostic and Wondfo biotech respectively and the second aliquot was stored at -20°C.

**Statistical analysis:** we used Microsoft Excel 2016 and transferred it to the Statistical Package for Social Sciences (SPSS) software version 22.0. The Chi-square test was computed for comparing proportions of EBV, HIV, HBV and HCV between groups of lymphomas. The statistical analysis was conducted with a 95% confidence interval and a p-value of <0.05 as a threshold of statistical significance.

**Ethical considerations:** the ethics committee of the Yaoundé General Hospital approved this study (*N/Ref: 1890/018/HGY/DG/DPM/NC-TR du 10 December 2018*). Patients gave their informed consent before being included in this study. Samples were anonymously screened, and no information made it possible to identify the patient.

## Results

From the 1^st^ October 2018 to 31^st^ December 2019, 91 patients with NHL were included in this study. Sixty-three were male (69.23%), and twenty-eight were female (30.77%). Their ages ranged from nineteen to seventy-eight years with a mean ± SD of 56.5 ± 15.5 (Table 1). Of the 91 patients with NHL, fifty-eight were married (63.7%) and thirty-three were single (36.3%). Concerning alcohol consumption, sixty-six patients (72.52%) were drinking before they fall sick. We recorded twelve patients (13.2%) who were smoking before their illness (Table 1). Thirty-four patients had diffuse large B cell lymphoma (DLBCL) (37.4%), fifteen patients had marginal zone lymphoma (MZL) (16.5%), thirteen patients had Burkitt lymphoma (BL) (14.3%), nine patients had either mantle cell lymphoma (MCL) or follicular lymphoma (FL) (9.9%), six patients had peripheral T cell lymphoma (PTCL) (6.6%) and five patients come with cutaneous T cell lymphoma (CTCL) (5.5%) (Table 1). Concerning the disease stage, thirty-two patients (35.2%) come at stage II of Ann Arbor classification followed by thirty patients (33%) at stage III, twenty-three patients (25.3%) and 6 patients (6.6%) come at stage IV and stage I respectively (Table 1). Regarding the viral infection, 8 patients (8.79%) had HIV followed by 5 patients (5.49%) with EBV or HBV. Three patients were coinfected with HIV+EBV (3.29%). While 2 patients (2.19%) had HCV. There was no statistical difference between groups compared at *p = 0.347* (Table 2).

**Table 1:** demographic and clinical characteristics of study participants recruited from the Medical Oncology unit of Yaoundé General Hospital (Cameroon) from Oct 2018 to Dec 2019 (N=91)

Variable		
Age	Frequency (%)	Mean ± S.D
< 60 years	40 (43.95)	56.5 ± 16
≥ 60 years	51 (56.04)	
**Gender**		
Male	63 (69.23)	
Female	28 (30.77)	
**Matrimonial status**		
Married	58 (63.7)	
Single	33 (36.3)	
**Alcohol consumption**		
Yes	66 (72.52)	
No	25 (27.47)	
**Smoking**		
Yes	12 (13.2)	
No	79 (86.8)	
**Type of lymphoma**		
Burkitt lymphoma	13 (14.3)	
Cutaneous T cell lymphoma	5 (5.5)	
Diffuse large B cell lymphoma	34 (37.4)	
Follicular lymphoma	9 (9.9)	
Mantle cell lymphoma	9 (9.9)	
Marginal zone lymphoma	15 (16.5)	
Peripheral T cell lymphoma	6 (6.6)	
**Stage of Ann Arbor**		
I	6 (6.6)	
II	32 (35.2)	
III	30 (33)	
IV	23 (25.3)	

**Table 2:** viral infections found in study participants with NHL recruited from the Medical Oncology unit of Yaoundé General Hospital (Cameroon) from Oct 2018 to Dec 2019 (N=91)

Viral infection	Frequency (%)	Chi-square test
EBV+	5 (5.5)	p = 0.347
HBV+	5 (5.5)	
HCV+	2 (2.2)	
HIV+	8 (8.8)	
HIV+ and EBV+	3 (3.3)	

EBV:; HIV: human immunodeficiency virus; HBV: hepatitis B virus; HCV: hepatitis C virus; +: positive

## Discussion

In this population-based cross-sectional study among NHL patients in the oncology unit of YGH, we consecutively interviewed 91 patients from 1^st^ October 2018 to 31^st^ December 2019. Firstly, results revealed the predominance of the elderly (> 60 years) and male participants, similar to previous study [2]. Also, 66% of patients were drinking alcohol and 12% of patients were smoking before they fell sick. There were controversial results about the association between alcohol, tobacco and NHL. Most reports have shown no excess risk of NHL overall in tobacco or cigarette smokers but there are several exceptions. Some studies have suggested a specific association with the risk of the common follicular NHL subtype [4]. Thirdly, the role of alcohol in the development of NHL is uncertain. Previous study of alcohol intake have shown no association or increased or reduced risk of NHL overall [1]. Fourthly, this study revealed that most patients were stage 2, followed by stage 3 and 4 of the Ann Abbor classification. Moreover, in Africa, endemic Burkitt´s lymphoma accounts for a substantial proportion (25-44%) of all NHL [2] but was under that proportion during this study. Furthermore, most non-Hodgkin lymphomas arise from mature B lymphocytes, with a minority derived from T lymphocytes. These results were previously described by study before [8]. Risks for developing NHL include immunosuppression and, a causal link between infectious agents, and lymphomagenesis has also been determined [2]. Here, 8.8% had HIV and 3.3% had coinfection HIV with EBV. EBV alone accounted for 5.5% since EBV is a herpes virus that is highly prevalent worldwide [9]. It shows an 80-90% seroconversion in young adulthood in most countries and is a major cofactor in many B-lymphoproliferative tumors [5]. Hepatitis B virus accounted of 5.5% in our study, and HCV had 2.2%, then Kang *et al*., were found a higher prevalence of HBV (12.4%) and for HCV, prevalence (2.8%) in Korean patients with hematopoietic malignancies [10] was similar to this study. Finally, the epidemiological association between HBV and HCV infection and non-Hodgkin´s lymphomas has been explored in populations of various countries and more recent evidence suggests a role of both viruses in NHL etiopathogenesis [5].

## Conclusion

Non-Hodgkin lymphoma is a lymphoid malignant neoplasm with multiple subtypes, and the most frequent is diffuse large B-cell lymphoma (DLBCL). It affects more males than females, and age the risk is >55 years. Our study suggests that EBV is an important cofactor in the development of NHL in HIV-infected persons and confirms previous reports. The presence of HBV and HCV in single infection, in patients with NHL reveals the need to understand how these viruses induce lymphoproliferative diseases. However, the possibility that NHL etiopathogenesis disorders could be caused by these viruses may have a significant effect on the overall disease burden due to its high prevalence in many populations. This could lead to a better understanding of the role played by all infectious agents in the pathogenesis of human tumors, more precisely, the non-Hodgkin´s lymphoma which might further lead to the development of new therapeutic strategies.

### What is known about this topic

- The non-Hodgkin's lymphomas affects more males than females;
- HIV/EBV coinfection is common among persons with non-Hodgkin's lymphomas.

### What this study adds

- The presence of HBV and HCV as single infection, in patients with non-Hodgkin's lymphomas.
